# Both localized and systemic bacterial infections are predicted by injection drug use: A prospective follow-up study in Swedish criminal justice clients

**DOI:** 10.1371/journal.pone.0196944

**Published:** 2018-05-31

**Authors:** Disa Dahlman, Jonas Berge, Per Björkman, Anna C. Nilsson, Anders Håkansson

**Affiliations:** 1 Lund University, Department of Clinical Sciences Lund, Division of Psychiatry, Lund, Sweden; Malmö Addiction Centre, Malmö, Sweden; 2 Department of Translational Medicine Malmö, Infectious Disease Research Unit, Lund University, Lund, Sweden; Rudjer Boskovic Institute, CROATIA

## Abstract

**Background:**

Both skin and soft tissue infections (SSTI) and systemic bacterial infections are common in people who inject drugs (PWID), but data on incidence and risk factors are lacking. We compared registered diagnoses for such infections in Swedish criminal justice clients with regard to injecting drug use.

**Methods:**

Baseline interview data from the Swedish Prison and Probation Service on drug use in PWID and non-PWID with problematic alcohol use were linked to follow-up data from national Swedish registers on hospital diagnoses and/or death. Associations between drug use and later diagnosis of SSTI and systemic bacterial infection (septicemia or bacterial infection of the heart, bone/joints or central nervous system) were analyzed by Cox regression.

**Results:**

Incidence rates of SSTI was 28.3 per 1,000 person-years for PWID (n = 2,444) and 10.0 for non-PWID with problematic alcohol use (n = 735). Incidence rates of systemic bacterial infection was 9.1 per 1,000 person-years for PWID and 2.7 per 1,000 person-years for non-PWID. Injection drug use was associated with a significantly increased risk of bacterial infections, for main drugs heroin (SSTI: Hazard ratio [HR] 2.45; systemic infection: HR 2.75), amphetamine (SSTI: HR 1.60; systemic infection: HR 2.19), and polysubstance use (SSTI: HR 1.92; systemic infection: HR 2.01). In relation to injection use of amphetamine and polysubstance use, PWID mainly using heroin had higher risk of SSTI.

**Conclusions:**

Injection drug use predicted both SSTI and systemic bacterial infection, with a particularly high risk of SSTI in PWID mainly using heroin. The results imply the need for increased attention to bacterial infections among PWID, in terms of clinical management, prevention and research.

## Introduction

Bacterial skin and soft tissue infections (SSTI) are among the most common reasons for people who inject drugs (PWID) to seek medical care [1–2]. Deep-seated, systemic infections such as septicemia, infective endocarditis [3–4], and infections in bone and joints [5] among PWID are considered complications of injection drug use in clinical practice, but this problem has mainly been described in case reports. Large-scale studies regarding incidence and predictors for such bacterial infections among PWID are lacking [1].

SSTI have been associated with injection of tissue irritating substances such as crushed tablets [6], black tar heroin [7], and speedball (a mix of heroin and cocaine) [2,8], but the association between SSTI incidence and the type of main drug has not been studied systematically.

In this study we present longitudinal data on SSTI and systemic bacterial infections among PWID where drug injection is a potential route of bacterial infection. The analysis did not include respiratory/urinary tract or gastrointestinal infections. For this purpose, we used registers from the Swedish criminal justice system assessing injection drug use, with linkage to national diagnostic registers. Among clients in the criminal justice system worldwide, up to 60% are dependent on illicit drugs [9] and up to 20% report a history of injection drug use [10]. Twenty-two percent of male and 41% of female Swedish inmates in the early 2000s have been diagnosed with drug use disorders [11]. Systematic data on the prevalence of injection drug use in Swedish prisoners have been reported only in older studies and from a limited number of study participants, but where Swedish lifetime prevalence rates of injecting (25 percent in a relatively small sample) were comparable to those of other European countries [12]. In contrast, the database on which the present study is based is likely to be the most systematic reporting of the extent of injection drug use in groups of criminal justice clients with problematic drug use in Sweden. In the present database, past-30-day injecting was reported by 63 and 70 percent of primary heroin and amphetamine users, respectively [13].

Specifically, we aimed to (1) investigate the incidence of SSTI, systemic bacterial infections, and fatal bacterial infections among PWID, and (2) analyze potential predictors of SSTI and systemic bacterial infections related to injection drug use, and injection use of specific substances in relation to each other.

## Materials and methods

### Study subjects

The current study includes subjects from the Swedish Prison and Probation Service, who completed an interview according to Addiction Severity Index (ASI) between 2001 and 2006. ASI is a well-documented interview instrument for assessment of substance use and substance-related problems in clinical settings and in the criminal justice system [14–15]. The ASI material consists of interviews from 7,085 individuals. Approximately 6% refused the ASI interview according to previous studies [16]. Fifty cases were excluded due to inadequate replies. The best available data describing elapsed time from intake to the criminal justice facility where the interview took place to the interview was 60 days (median 27 days; 98% were interviewed within one year). Baseline data from the ASI database include information regarding substance use, medical conditions, psychiatric health and demographic variables, which have been used in previous longitudinal studies [17].

**Fig 1:**
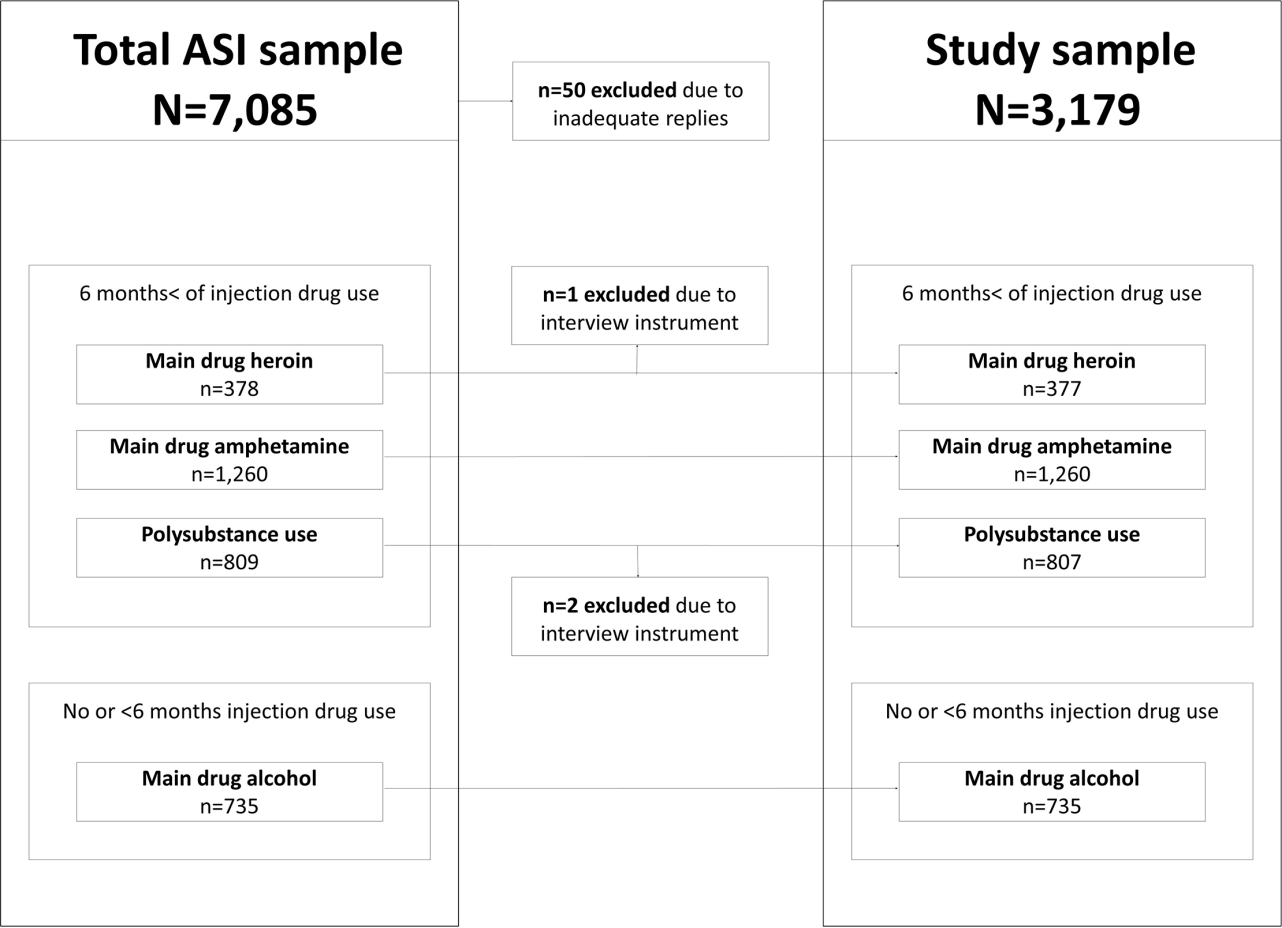
Selection of study participants from the Addiction Severity Index (ASI) database.

Subjects from the ASI database were included in the study if they either had reported regular injection drug use *and* main drug heroin, amphetamine or polysubstance use (two or more main drugs, of which at least one illicit drug); or if they had denied regular injection drug use *and* reported alcohol as main drug (Fig 1). Regular injection drug use was defined as self-reported regular injection drug use for at least 6 months, prior to admission to the criminal justice facility. Main drug was assessed through the ASI multiple choice question “Which substance is the major problem?” For individuals reporting regular injection drug use the type of main drug (heroin, amphetamine or polysubstance use) was registered. Three cases were excluded because another interview instrument than ASI was used.

### Covariates and outcome measures

All covariates were retrieved from the ASI database, and included sex, self-reported homelessness past 30 days, residence in a major city (population >100,000), self-reported drug injection past 30 days, self-reported previous overdose, self-reported hepatitis C. Incarceration was also included as a control variable.

Follow-up data on infection outcomes and mortality were collected from two Swedish national health registers: the National Patient Register (NPR) and the Causes of Death Register (CDR). Information on bacterial infections was extracted from the NPR for hospital-based inpatient and outpatient care in which physicians’ diagnoses according to International Classification of Diseases 10 (ICD-10) are registered [18]. Causes and dates of deaths were collected from the CDR held by the Swedish National Board of Health and Welfare [19]. The CDR includes all deceased individuals who were registered in the Swedish Population Register at the time of death between 1961 and 2011. From 2012 the CDR includes all individuals deceased in Sweden, including individuals not registered in the Swedish Population Register. Ultimate and contributory causes of death are registered by physicians as diagnostic codes according to ICD in a death certificate.

The outcome variables retrieved from the NPR included the following main and secondary ICD-diagnoses: Infections in skin or soft tissue (ICD-codes L00–L03, L08, A46), cardiac infections (ICD-codes I30.1, I32.0, I33, I38, I39), infections in joints, skeleton or muscles (ICD-codes M00, M46.2, M46.3, M46.5, M60.0, M65.0, M65.1, M86, A48.0), intracranial and intracolumnar infections (ICD-codes G00.3, G04.2, G06, G07), and septicemia (ICD-codes A40, A41.0–A41.2). ICD-codes for infections in the respiratory/urinary tract or the gastrointestinal system were not included. Outcome variables from the CDR included all registered deaths during follow-up, where the ultimate or contributory cause of death was any of the ICD-10 diagnoses above.

The Prison and Probation Service and the Swedish National Board of Health and Welfare linked ASI data with diagnosis data from 2001–2014 through national identification numbers. Data delivered to the researchers were de-identified. All data were accessed in 2016. Data retrieval was preceded by an opt-out procedure, through notification of the study in a free magazine in the three major Swedish urban areas (Stockholm, Gothenburg and Malmö). No subjects chose to opt out. The study was approved by Lund Regional Ethics Board (file number 2014/478).

### Statistical analysis

For the multivariable time-to-event analyses, we used extended Cox regression models using incarceration (including time in custody) as a time-varying covariate. The reason for this was that the baseline assessments were in most cases performed at the start of the prison sentence, so the follow-up data for each individual reflect both the time in prison and the time after release from prison, until the first episode of SSTI, any systemic infection according to our study definitions, or death, respectively. We used a robust estimator of the standard errors to adjust for the double rows of data for each individual that served a prison sentence during the study period. The time-scale used was the age of the study participants, which may be more important than time-on-study in observational studies [20].

Cox regression analysis was conducted with the outcome variables 1) SSTI during follow-up, and 2) Systemic infection during follow-up. All systemic infections were collapsed into one variable. In order to identify both potential associations between injection drug use and infections compared to non-injection, and potential differences between injection use of certain substances, we conducted two Cox regression models with different reference categories: Non-injection drug use with alcohol as main drug, and injection drug use with heroin as main drug, respectively.

Prior to the analyses, we performed a partial model selection for each of the Cox regression models. Based on the literature, we defined the variables main drug [2,7–8], sex [8,21], homelessness at baseline [6] and the time-varying variable incarceration as being of primary importance. The other variables (i.e. injection drug use in the past 30 days at baseline, living in a metropolitan area, self-reported hepatitis C and previous overdose) were defined as being of secondary importance. The variables of primary importance were a priori included in all analyses whereas the variables of secondary importance were subjected to a backward elimination procedure where, at each step, the variable with the highest non-significant p-value in a series of F-tests (each using a chi squared distribution with one degree of freedom) was removed from the model. This procedure was repeated until none of the remaining variables of secondary importance could be removed without weakening the model significantly. The model selection process resulted in removal of injection drug use past 30 days as a predictor for SSTI; and injection drug use past 30 days, being resident in a large city, and previous overdose as predictors for systemic infection. None of the variables included in the two Cox regression models showed significant interactions between the scaled Schoenfeld residuals and the logarithm of time at the 0.05 level, indicating that there were no violations of the proportionality assumption in either model.

We assessed all independent variables for multicollinearity. The highest bivariate correlation was -0.49, between main drug amphetamine and polydrug use as main drug, which is expected because the main drug categories were mutually exclusive. The highest bivariate correlation except for between the main drug categories were 0.29 (between main drug alcohol and hepatitis C). The calculated variance inflation factor (VIF) was highest for alcohol as main drug at 3.63, which again is expected because the mutual exclusiveness among the main drug categories. The highest VIF among the remaining variables was 1.44 (for hepatitis C). All measures of multicollinearity were thus well within acceptable limits.

Data preparation and statistical analyses were performed in R 3.3.2 [22]. The package 'survival' was used for estimating the Cox regression models [23]. Ten individuals were excluded from statistical analyses due to missing information about age in the baseline interview.

## Results

### Population characteristics

A total of 3,179 subjects (14% female, mean age 36.1 years [standard deviation 10.0 years], 77% PWID) were included in the study (Table 1). Self-reported main drug was amphetamine in 40%, polysubstance use in 25%, heroin in 12%, and alcohol in 23%. Almost 25% reported being homeless prior to baseline. Hepatitis C was reported by 62%. Median and total follow-up time, respectively, was 9.8 years and 27,805 person-years for the outcome variable SSTI; 10.2 years and 30,175 person-years for systemic infection; and 10.3 years and 31,196 person-years for death.

**Table 1:** Baseline characteristics. Baseline characteristics among 3,179 criminal justice clients with history of either injecting drug use (PWID) or problematic alcohol use without injecting drug use (non-PWID). Baseline data from Addiction Severity Index interview.

Variable	Total samplen (%)	PWID^i^n (%)	Mainly heroinn (%)	Mainly ampheta-minen (%)	2≤ main drugsn (%)	Non-PWID^ii^n (%)
Study participants	3,179 (100)	2,444 (100)	377 (100)	1,260 (100)	807 (100)	735 (100)
Prison or custody	2,608 (82)	2,120 (87)	295 (78)	1,099 (87)	726 (90)	488 (66)
Female sex	429 (14)	342 (14)	52 (14)	205 (16)	85 (11)	87 (12)
Homeless past 30 days	790 (25)	715 (29)	75 (20)	374 (30)	266 (33)	75 (10)
Resident in a large city (population >100,000)	1,311 (41)	1,057 (43)	221 (59)	505 (40)	331 (41)	254 (35)
Self-reported hepatitis C	1,962 (62)	1,916 (78)	308 (82)	1,005 (80)	603 (75)	46 (6)
Main drug						
Alcohol	735 (23)	0	0	0	0	735 (100)
Heroin	377 (12)	377 (15)	377 (100)	0	0	0
Amphetamine	1,260 (40)	1,260 (52)	0	1,260 (100)	0	0
Polysubstance use	807 (25)	807 (33)	0	0	807 (100)	0
Drug injection past 30 days	1,786 (56)	1,773 (73)	246 (65)	950 (75)	577 (72)	13 (2)
Previous overdose	1,088 (34)	1,044 (43)	241 (64)	380 (30)	423 (52)	44 (6)
Mean age in years	36.1	36.1	32.5	38.3	34.2	36.1

i = main drug heroin, amphetamine and polydrug use

ii = main drug alcohol

### Incidence of SSTI and systemic bacterial infections

Person-time incidence rates of SSTI were 28.3 per 1,000 person-years for PWID and 10.0 per 1,000 person-years for non-PWID (Table 2), with the highest incidence rate (41.5 per 1,000 person-years) among PWID reporting heroin as main drug. Incidence rates of systemic bacterial infection, according to our study definitions, were 9.1 per 1,000 person-years for PWID and 2.7 per 1,000 person-years for non-PWID. Incidence rates among PWID were 2.6 per 1,000 person-years for cardiac infection, 5.4 per 1,000 person-years for bone/joint infection, 0.6 per 1,000 person-years for CNS infection, and 3.6 per 1,000 person-years for septicemia.

**Table 2:** Incidence of bacterial infections. Incidence of skin and soft tissue infection (SSTI), systemic bacterial infections and fatal bacterial infections during follow-up 2001–2014. N = 3,179. Data retrieved from the National Patient Register and Causes of Death Register. Person-time incidence rates expressed as number of events per 1,000 person-years. Total follow-up time to SSTI: 27,805 person-years (median 9.8 years), to systemic infection: 30,175 person-years (median 10.2 years), and to death 31,196 person-years (median 10.3 years).

Variable	Total sample	PWID^i^	Mainly heroin	Mainly ampheta-mine	2≤ main drugs	Non-PWID ^ii^
Study participants (n)	3,179	2,444	377	1,260	807	735
SSTI^iii^	23.7	28.3	41.5	24.0	29.7	10.0
Systemic infection^iv^	7.6	9.1	10.4	9.5	8.0	2.7
Cardiac infection^v^	2.0	2.6	2.9	2.6	2.3	0.3
Bone/joint infection^vi^	4.7	5.4	7.3	5.4	4.7	2.1
CNS infection^vii^	0.5	0.6	0	0.7	0.8	0.1
Septicemia^viii^	3.0	3.6	4.3	3.8	3.1	0.7
Fatal bacterial infection	0.6	0.7	0.6	1.0	0.4	0.3
Overall mortality	14.7	14.7	21.8	12.4	15.3	14.6

i = main drug heroin, amphetamine and polydrug use

ii = main drug alcohol

iii = ICD-codes L00–L03, L08, A46

iv = any of the ICD-codes [iii] or [v-viii]

v = ICD-codes I30.1, I32.0, I33, I38, I39

vi = ICD-codes M00, M46.2, M46.3, M46.5, M60.0, M65.0, M65.1, M86, A48.0

vii = ICD-codes G00.3, G04.2, G06, G07

viii = ICD-codes A40, A41.0–A41.2

### Predictors of SSTI and systemic bacterial infections

In multivariable analysis, injection drug use with main drug heroin, amphetamine or multiple substances was significantly associated with SSTI in the first Cox regression model (Table 3). Homelessness, residence in a large city, previous overdose, and hepatitis C was associated with increased risk of SSTI. Incarceration was associated with lower risk of SSTI. The second Cox regression model showed that amphetamine and polysubstance use was associated with lower risk of SSTI, when using heroin as the reference category.

**Table 3:** Associations with skin and soft tissue infection (SSTI) during follow-up. Cox regression analysis with non-injection (Model 1) and heroin injection (Model 2) as reference categories. Time measured from baseline interview to SSTI diagnosis. N = 3,169. Number of events = 660.

Predictor variable	Model 1 (non-injection vs injection)Hazard ratio (95% CI)	Model 2 (heroin vs other main drug)Hazard ratio (95% CI)
Main drug (mutually exclusive)		
Alcohol (= non-injectors)	1 [reference]	0.41 (0.28–0.59)***
Heroin	2.45 (1.70–3.52)***	1 [reference]
Amphetamine	1.60 (1.16–2.20)**	0.66 (0.52–0.83)***
Polysubstance use	1.92 (1.39–2.65)***	0.79 (0.62–1.00^i^)*
Female sex	0.87 (0.69–1.10)	0.87 (0.69–1.10)
Homeless 30 days prior to baseline	1.23 (1.04–1.46)*	1.23 (1.04–1.46)*
Resident in large city	1.37 (1.17–1.60)***	1.37 (1.17–1.60)***
Previous overdose	1.39 (1.18–1.64)***	1.39 (1.18–1.64)***
Self-reported hepatitis C	1.43 (1.15–1.77)**	1.43 (1.15–1.77)**
Incarceration	0.61 (0.47–0.80)***	0.61 (0.47–0.80)***

i = significant association, upper limit of confidence interval rounded off to two decimals

* p<0.05

** p<0.005

*** p<0.001.

Predictors of systemic infection in the first Cox regression model were injection drug use with main drug heroin, amphetamine and polysubstance use (Table 4). Homelessness and hepatitis C was associated with increased risk of systemic infection, and incarceration was associated with decreased risk. The second Cox regression model did not show significant differences between injection use of heroin, amphetamine and polysubstance use, with regard to systemic infection.

**Table 4:** Associations with systemic bacterial infection during follow-up. Cox regression analysis with non-injection (Model 1) and heroin injection (Model 2) as reference categories. Time measured from baseline interview to systemic bacterial infection diagnosis (collapsed variable). N = 3,169. Number of events = 230.

Predictor variable	Model 1(non-injection vs injection)Hazard ratio (95% CI)	Model 2(heroin vs other main drug)Hazard ratio (95% CI)
Main drug (mutually exclusive)		
Alcohol (= non-injectors)	1 [reference]	0.36 (0.19–0.71)**
Heroin	2.75 (1.41–5.39)**	1 [reference]
Amphetamine	2.19 (1.20–4.02)*	0.80 (0.54–1.17)
Polysubstance use	2.01 (1.07–3.76)*	0.73 (0.48–1.11)
Female sex	0.83 (0.56–1.22)	0.83 (0.56–1.22)
Homeless 30 days prior to baseline	1.35 (1.03–1.78)*	1.35 (1.03–1.78)*
Self-reported hepatitis C	1.58 (1.04–2.40)*	1.58 (1.04–2.40)*
Incarceration	0.36 (0.19–0.66)**	0.36 (0.19–0.66)**

* p<0.05

** p<0.005.

### Fatal bacterial infections

During follow-up, overall mortality was 14.7 per 1,000 person-years among PWID and 14.6 per 1,000 person-years among non-PWID, but fatal bacterial infections were rare causes of death in both groups (<1.0 per 1,000 person-years; Table 3). Due to a small number of infection related fatalities, multivariable analysis was not conducted with this outcome variable.

## Discussion

In this study of clients in the Swedish criminal justice system we found high incidence rates of both SSTI and systemic bacterial infections among PWID, and both of these were significantly associated with injecting drug use. For diagnoses where data is available for the general Swedish population, incidence rates among PWID were remarkably high. In the case of infective endocarditis, the incidence among PWID in this study was more than 30 times higher than the average incidence in Sweden 1997–2007 [24]. To our knowledge, this is the first large-scale, longitudinal register study focusing on the epidemiology of bacterial infections among people who use drugs.

A suggested reason for the high infection rates among PWID has been abundant bacterial colonization with *Staphylococcus aureus* in combination with repeated skin lesions [25]. High injection frequency has also been associated with SSTI [8,21] further supporting bacterial transfer from the skin as an important route of infection. For these reasons, the systemic bacterial infections assessed in this study were selected based on their potential association with bacterial transfer from the skin through injection drug use. Future longitudinal studies with bacteriological testing could help to clarify associations and mechanisms involved between bacterial colonization and infections among PWID.

We observed that use of heroin, compared to other injectable drugs, was significantly associated with SSTI. Use of black tar heroin is described as a cause or predictor of SSTI in the U.S. [7]. Our findings are in agreement with those reported from Colorado, USA, with higher rates of SSTI among heroin users compared to subjects using methamphetamine and cocaine [26]. In Sweden, where black tar heroin use is extremely rare, people injecting heroin have been shown to be younger than amphetamine and polysubstance users, have higher mortality rates, less stable housing, more criminal involvement and are more often involved in commercial sex [13,27]. In a Swedish setting, this association might rather be explained by more severe substance dependence and more vulnerable socio-economic situation among people injecting heroin.

Apart from heroin use, reported history of overdose, being resident in a large city, homelessness and self-reported hepatitis C were independently associated with increased risk of SSTI. Homelessness and hepatitis C were also associated with increased risk of systemic infection. Unstable housing, overdose and hepatitis C among PWID have been linked to more risky injection practices such as needle sharing and street injection [28–30], which likely also leads to a higher risk of bacterial infections. In a prospective longitudinal study from a Swedish NEP, Alanko Blomé et al identified injection use of amphetamine (in contrast to heroin) to be significantly associated with lower odds of hepatitis C virus seroconversion [31]. The finding that incarceration was associated with lower risk of SSTI and systemic infection may be explained by better access to healthcare and hygiene.

The proportion of deaths due to infection was rather low in this population, which is probably due to high overall mortality through unnatural causes such as intoxication, accidents and suicide [17]. In a longitudinal study based on Swedish register data, overall mortality after release from prison was 6% during a median of 5 years follow-up time, with significantly increased risk of death among previously incarcerated people with alcohol or drug dependence [11]. The non-PWID group of the present study constituted clients with primary alcohol problems, and although beyond the scope of the present study, the comparably high rates of mortality in non-PWID, compared to the PWID group as a whole, is likely to be directly or indirectly related to alcohol-related complications. Our study design did not allow for assessment of clinical consequences and outcomes of bacterial infections recorded.

The findings in this study demonstrate the need for heightened awareness towards bacterial infections in PWID. Bacterial infections might be prevented through improved skin and injection hygiene [32] but evidence-based data on relevant interventions is needed. Since sharing of injection equipment has been shown to increase likelihood of SSTI 5–6 fold [33], access to clean injection equipment for PWID can probably also help reduce the incidence of SSTI. The findings that injection drug use in general, and injection use of heroin in particular, was associated with increased risk of bacterial infections suggest that opioid substitution treatment (OST) could prevent these common health hazards among PWID. OST participation and retention have been shown to be associated with improved quality of life [34] and improved self-perceived physical health [35] among people with opioid dependence. During the years 2001–2006 when the data collection was conducted, access to clean injection equipment as well as OST was limited. There were only two needle exchange facilities in Sweden until 2010. Prior to 2005, OST participation was limited to PWID with at least four years of documented intravenous opioid use, and at least three failed attempts at drug-free treatment [36]. Since the harm reduction situation in Sweden has changed dramatically, particularly regarding OST access, the results might have been different if the study was repeated.

This study has some limitations. No comparative analyses regarding socioeconomic conditions among PWID with different drug use patterns were possible. Injection frequency, injection habits, periods of abstinence and living conditions during follow-up are likely to affect the infection prevalence [2,6,8,21,26,33]. Potentially strong predictors of bacterial infections not included in this study are HIV infection and diabetes mellitus. While HIV has been associated with SSTI and endocarditis [8], we did not include self-reported HIV in the analyses, since self-reported HIV infection is extremely rare in the ASI material (personal communication, A. Håkansson).

The category systemic bacterial infection was analyzed as a collapsed variable based on several groups of ICD-diagnoses where transfer of bacteria from the skin in connection to injection is a probable route of infection. A larger number of cases would have allowed more refined analyses of specific bacterial infections, also with regard to bacteriological etiology.

While a large percentage of criminal justice clients are using drugs according to international [9–10] and Swedish data [11], our study population might not be representative for PWID in the community. It is plausible that PWID in the criminal system have worse health status, as well as higher mortality than others [10–11]; however, high proportions (47%) of PWID attending a NEP in our uptake area report imprisonment [31], and demographic data were comparable with data from this NEP regarding distribution of sex, age and main drug [31,37].

Strengths of the study are the large number of included individuals, and use of register data of high validity. The ASI data are based on self-reports, but has been shown to have high reliability in previous research [38–39]. In the National Inpatient Register, main diagnosis is registered in 99% of inpatient episodes [18], and 85–95% of diagnoses were valid when comparing with inpatient records [40]. In the CDR, 96.5% of all fatalities have information regarding causes of death [19], with higher reliability [41] in younger individuals and in fatalities due to unnatural causes of death (in which autopsies are routinely performed). The use of criminal justice clients with problematic alcohol use, rather than healthy subjects, as reference group, decreases the risk of selection bias based on socioeconomic status and incarceration. There is, however, a possibility of misclassification, since we did not have access to data on main drug or regular injection drug use during follow-up.

## Conclusions

In this study, we show that PWID have significantly increased risk of SSTI or systemic bacterial infections, compared to individuals with problematic alcohol use, with a particularly high risk of SSTI among heroin users. These findings imply the need for increased attention to bacterial infections among PWID, both with regard to clinical management, prevention and research.

## Supporting information

S1 File ICD-diagnoses supplement. (DOC)

## Abbreviations

ASI: Addiction Severity Index
CDR: the Causes of Death Register
HR: hazard ratio
ICD: International Classification of Diseases
NEP: needle exchange program
NPR: the National Patient Register
OST: opioid substitution treatment
PWID: people who inject drugs
SSTI: skin and soft tissue infections
VIF: variance inflation factor
