# Supplementary material for: Both localized and systemic bacterial infections are predicted by injection drug use: A prospective follow-up study in Swedish criminal justice clients
Source: PLoS One. 2018 May 31;13(5):e0196944. doi: 10.1371/journal.pone.0196944 (PMC5979029; doi:10.1371/journal.pone.0196944)
Supplement: S1 File — (DOC) [file pone.0196944.s001.doc]

**Supplement: Diagnoses according to ICD-10**

**Skin and soft tissue infection**

L00 Staphylococcal scaled skin syndrome

L01 Impetigo

L02 Cutaneous abscess, furuncle and carbuncle

L03 Cellulitis

L08 Other local infections of skin and subcutaneous tissue

A46 Erysipelas

**Cardiac infection**

I30.1 Infective pericarditis

I32.0 Pericarditis in diseases classified elsewhere

I33 Acute and subacute endocarditis

I38 Endocarditis, valve unspecified

I39 Endocarditis and heart valve disorders in diseases classified elsewhere

**Bone/joint/muscle infection**

M00 Pyogenic arthritis

M46.2 Osteomyelitis of vertebra

M46.3 Infection of intervertebral disk (pyogenic)

M46.5 Other infective spondylopathies

M60.0 Infective myositis

M65.0 Abscess of tendon sheath

M65.1 Other infective (teno)synovitis

M86 Osteomyelitis

A48.0 Gas gangrene

**Central nervous system infection**

G00.3 Staphylococcal meningitis

G04.2 Bacterial meningoencephalitis and meningomyelitis, not elsewhere classified

G06 Intracranial and intraspinal abscess and granuloma

G07 Intracranial and intraspinal abscess and granuloma in diseases classified elsewhere

**Septicemia**

A40 Streptococcal septicaemia

A41.0 Septicaemia due to Staphylococcus aureus

A41.1 Septicaemia due to other specified staphylococcus

A41.2 Septicaemia due to unspecified staphylococcus
